# Experimental and Numerical Modeling of Liposome Congregation in Meteorite Craters of Early Earth

**DOI:** 10.3390/life16040542

**Published:** 2026-03-25

**Authors:** Vladimir M. Subbotin, Benjamin A. Turner, Brian A. Davies, Alric G. Lopez, Gennady Fiksel

**Affiliations:** 1 Arrowhead Pharmaceuticals Inc., Madison, WI 53719, USA; 2 Department of Physics, University of Wisconsin, Madison, WI 53706, USA; gfiksel@wisc.edu

**Keywords:** origin of life, liposomes, Darwinian evolution, meteor craters, earthquakes

## Abstract

This paper provides experimental and numerical evidence supporting the occurrence of liposome congregation at the floors of meteor craters on Early Earth. This work builds on our earlier research, which demonstrated that liposomes submerged in a shallow Archean pond are protected from harmful UV radiation. This protection enables them to survive sufficiently long for autocatalytic amphiphile replication and for the mutation and selection of assemblies that enhance membrane stability. For liposomes to fuse, grow, exchange contents and membrane components, and divide, they must establish a population, i.e., form a dense conglomerate that enables close physical contact. The study demonstrates that such a congregation is feasible in bowl-shaped meteor craters on Early Earth, especially under periodic seismic disturbances.

## 1. Introduction

In our earlier publication [1], we proposed a hypothesis on the Darwinian evolution of liposomes based solely on natural and ever-present phenomena: the diurnal cycle of solar UV radiation, gravity, and the formation and release of amphiphiles in aqueous media. The hypothesis is based on the premise that amphiphilic molecules introduced into Archean water inevitably accumulate at the water–air interface and form Langmuir layers, bilayers, and liposomes.

In our scenario, the newly formed liposomes would inevitably be destroyed by solar UV unless they acquire negative buoyancy by capturing heavy solutes, such as ribose, and descend from the water–air boundary. We have also shown that some primordial water constituents, such as ferric salts, can provide UV attenuation sufficient to shield liposomes at depths of only a few millimeters [2,3]. The submerged liposomes, shielded from damaging UV radiation, acquire the longevity necessary to enhance autocatalytic replication of amphiphiles, their mutation, and the selection of those amphiphilic assemblies that provide the greatest membrane stability. These two types of mutable and heritable compositional information, heavy content and amphiphilic assembly design, constitute adaptive traits influenced by natural selection. Selection and propagation of the best-fitted traits drive Darwinian evolution.

However, liposome survival alone, while necessary, is not sufficient to drive Darwinian evolution. For that, the surviving liposomes must form a population [4,5,6,7]. Contemporary analyses emphasize that a population, or the formation of groups of similar entities, such as protocells, arising through temporal and spatial congregation, is fundamental to the origin of life [8,9]. Multiple in vitro studies showed that liposomes, when subjected to physical contact with each other, undergo fusion and growth [10,11,12,13], exchange of liposomal content and membrane components [14,15,16,17], and fusion and division [18,19,20]. Therefore, liposomes need to gather and create a population to undergo Darwinian evolution. This study explores how a liposomal population could form if liposomes are simply dispersed on the floor of primordial ponds.

We propose that meteorite craters on Hadean Earth may have served as sites for the self-sustaining Darwinian evolution of liposomes. There are other possible localisations for concentrating amphiphiles, such as freshwater hot springs, wet–dry cycles, evaporative pools, and closed-basin lakes. Presently, these environmental features can be found in Yellowstone National Park, Iceland’s geothermal fields, New Zealand’s geothermal zones, Kamchatka, and a few other sites. It remains unclear how common such fields were during the time of life’s emergence.

We believe that environments conducive to abiogenesis should be widespread rather than confined to specific areas. Evidence from the surfaces of the Moon and Mars suggests that 3.8 billion years ago, Earth’s surface was entirely covered with impact craters. Our meteorite hypothesis satisfies at least three key prerequisites. First, craters could host primordial amphiphiles, phospholipid precursors, and ribose, all of which could have been delivered or even generated by the meteorite impact. Second, filled with mineral-rich water, they could offer essential protection from solar UV radiation. Third, soil movement could help concentrate the biomaterials at the crater’s center and promote the formation of a population.

In support of our hypothesis, we mention that various studies [21,22,23,24] demonstrated that sites of meteorite impacts could be seeded with amphiphiles, phospholipid precursors, and ribose [25,26,27,28], the molecules that are essential for our hypothesized origin of life. More so, a recent experimental study demonstrated that the meteorite impacts themselves produced phospholipid precursors directly from Earth’s soil, in both wet and dry conditions [24]. Therefore, it is plausible to consider that meteorite craters on Hadean Earth could have been potential sites for the self-sustained Darwinian evolution of liposomes [29,30].

We have already demonstrated the effect of UV shielding by ferric salts [2,3]. But what about forming a population?

Meteorite craters, as shown by surface images of the Moon and Mars, exhibit a wide range of shapes and sizes [30]. Generally, their topography consists of concave, bowl-shaped depressions, dips, and crevices at scales ranging from tens of kilometers to meters and less. Each of these features, filled with water enriched with various minerals, particularly ferric salts, can serve as a site for liposome formation, survival, and growth. Although the concave shape of the sites theoretically encourages heavy liposomes to settle at the bottom by minimizing gravitational potential energy, this process is often impeded by friction and physical obstacles. However, external disturbances, such as local earthquakes and shock waves produced by meteorite bombardment, may shift the soil, thereby releasing liposomes and facilitating their downward migration. To investigate this issue and demonstrate the proposed mechanism, or at least its proof of principle, we created experimental and numerical models of liposomal behavior in meteorite craters undergoing sudden, repetitive displacements.

The paper is organized as follows. Section 2 describes the experimental setup and results. Section 3 describes numerical simulations of the experiments. Section 4 summarizes the results.

## 2. Experiment

### 2.1. Earthquake Data from the Literature

The experiment described in this section was designed and built to closely simulate the ground motion characteristics observed during earthquakes, such as the soil displacement magnitude and velocity. Detailed earthquake data, including amplitude and frequency spectra of various tremor types, can be found in [31]. Displacement measurements, assembled from data across multiple earthquake sites, vary from centimeters to tens of centimeters, while velocity values vary from a fraction of 1 m/s to several m/s. The experiment and numerical simulations were designed to match these characteristics.

### 2.2. Experimental Setup

**Figure 1:**
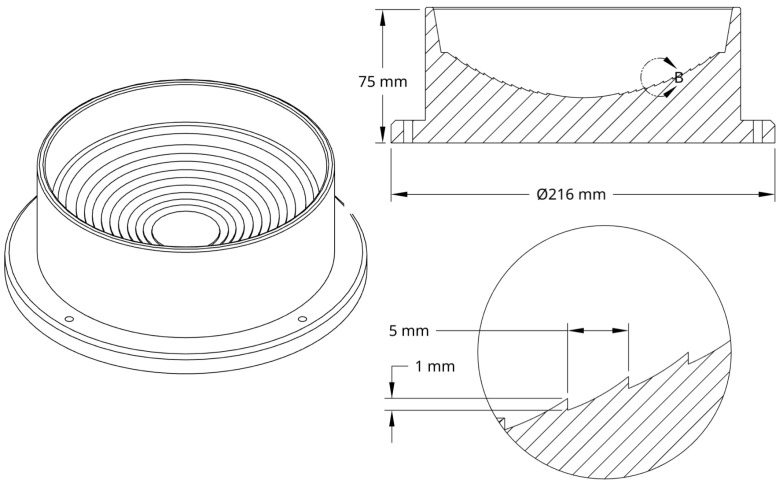
A drawing of a crater model showing an overview and detail views.

A “crater” model drawing is shown in Figure 1. The crater has a spherical cap shape with a sphere radius of 200 mm, a cap radius of 80 mm, and a height of 35 mm. It was produced using water-tight 3D printing technology. The base of the crater features ridges spaced every 5 mm, each measuring 1 mm in height. The crater is filled with water into which polyethylene red liposome-mimicking microspheres (Cospheric LLC, Somis, CA, USA), with a diameter of d=350 μm and density of $\rho =1.2\;g/{\mathrm{cm}}^{3}$, are submerged. The purpose of the ridges is to provide physical obstacles preventing the microspheres from rolling down the hill under gravity. Clearly, neither the size and shape of the “crater” nor the size, material, and mass of the “liposomes” approach their real-life counterparts. Rather, the purpose of this experiment is a demonstration of a general principle of liquid-submerged micro-particulate dynamics, driven by gravitation and assisted by periodic disturbances. In addition, comparing the experimental and numerical results will validate the numerical model and provide a basis for further development and improvement.

**Figure 2:**
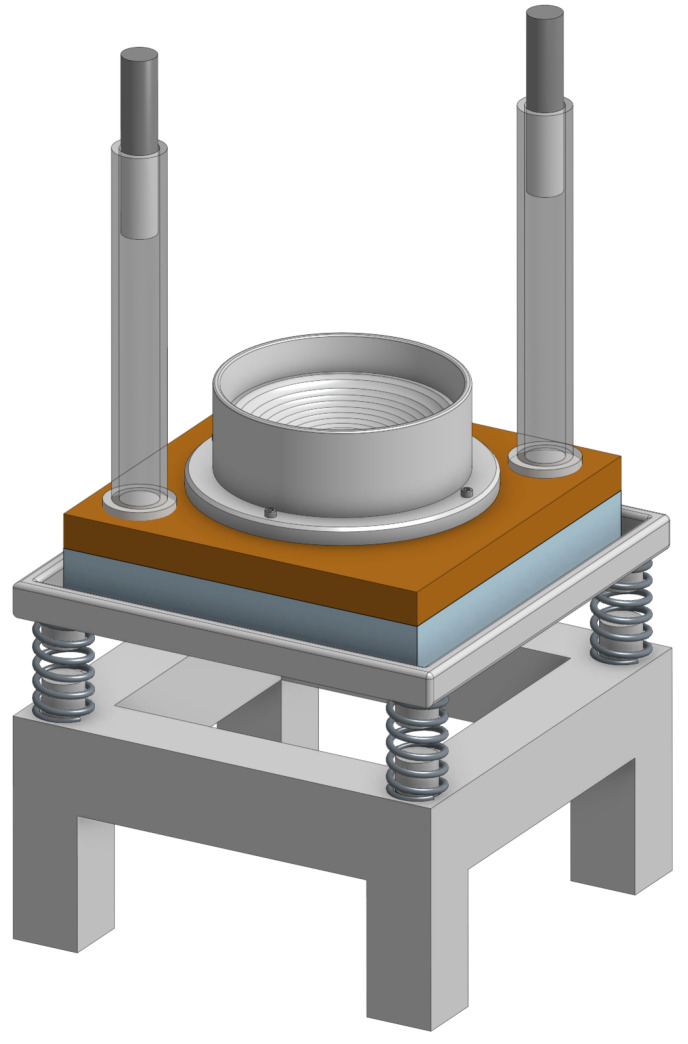
Experimental setup. A stainless steel tray is connected to a stand with four springs. The crater model is mounted on a 2 cm thick wooden plate, which is placed on top of a 3 cm thick polyurethane foam layer resting within the tray. Two drop weights, guided by two plastic cylindrical enclosures, are dropped from h=25 cm.

An experimental setup for investigating liposome behavior is shown in Figure 2 and consists of a stainless steel tray connected to a stand with four springs. The crater model is mounted on a 2 cm thick wooden plate, which rests on a 3 cm thick polyurethane foam layer within the tray. Two drop weights, guided by two plastic cylindrical enclosures, are dropped from h=25 cm, thereby generating the crater’s displacement through momentum transfer, thus emulating an initial quake jolt.

Since the foam offers minimal resistance at the initial moment, the crater velocity at impact can be estimated using momentum conservation:

(1) $$V_{c}=V_{w}\frac{M_{w}\left(1+e\right)}{M_{w}+M_{c}+M_{b}},$$

where $M_{w}$, $M_{c}$, and $M_{b}$ are the masses of the drop weights, the crater, and the board, respectively, and $V_{w}$ is the drop weight velocity at the collision. The coefficient of restitution (COR) *e* is the measure of the elasticity of collision. The higher *e* is, the more elastic the collision is.

As measured, $M_{c}+M_{b}=1.9\;kg$, with a calculated value of $V_{w}=\sqrt{2gh}=2.2\;m/s$. Two kinds of drop weights were used—steel bars with a total mass of $M_{w}=2.4\;kg$, and aluminum bars with a total mass of $M_{w}=0.83\;kg$. The COR depends on many factors, such as the materials, the impact velocity, and the shape of the colliding objects. To measure it directly, we repeatedly dropped the weights and measured their rebound heights. The only difference from the described setup was that the board was rigidly attached to a sturdy table. The weights’ motion was videoed at 240 fps.

In the experiment, at a drop height of H=250 mm, the measured rebound heights were h=24mm for the steel bars and h=32mm for the aluminum bars. The COR was calculated using $e=\sqrt{h/H}$, resulting in e=0.31 for steel bars and e=0.36 aluminum bars. Applying Equation (1) results in the velocity of the crater at the impact being $V_{c}=1.57\;m/s$ for the steel and $V_{c}=0.91\;m/s$ for the aluminum. The impact velocity figures align well with the earthquake data presented in Section 2.1.

### 2.3. Experimental Results

**Figure 3:**
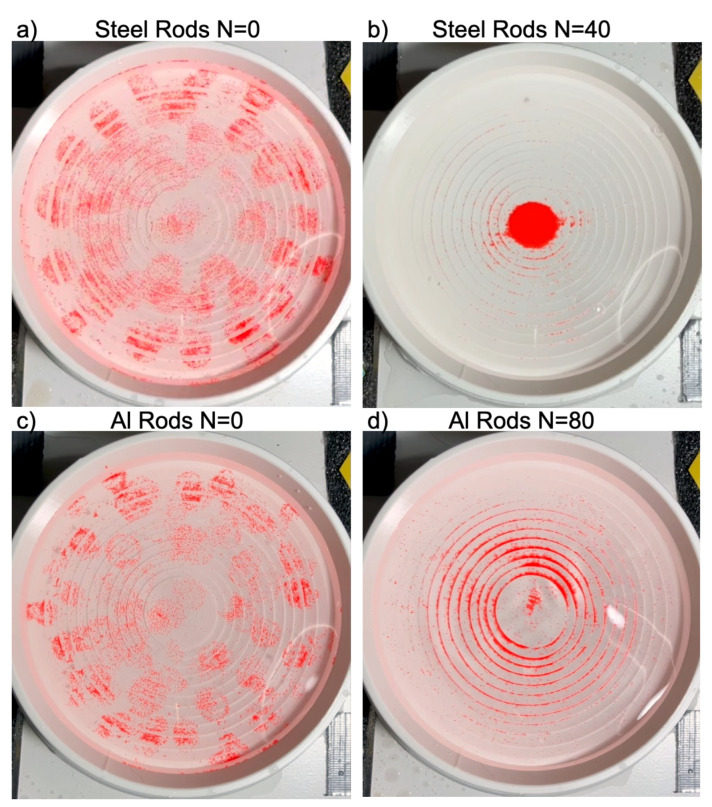
Summary of particle dynamics. The top and bottom rows compare results for steel and aluminum rods, respectively, while the left and right columns show the initial and final stages. (**b**) Dropping steel bars led to nearly complete particle collection at the center after N = 40 drops. (**d**) Dropping aluminum bars resulted in a profile that, although somewhat peaked, remained noticeably wide even after 80 drops.

Before each test, the red microspheres were spread evenly across the entire crater. Afterwards, a series of repeated weight drops was performed, and the movement of the particles was recorded on video. The results are summarized in Figure 3. The top and bottom rows compare results for steel and aluminum rods, respectively, while the left and right columns show the initial and final stages. Dropping steel bars led to nearly complete particle collection at the center after N = 40 drops. Dropping aluminum bars resulted in a profile that, although somewhat peaked, remained noticeably wide even after 80 drops.

## 3. Numerical Simulations

**Figure 4:**
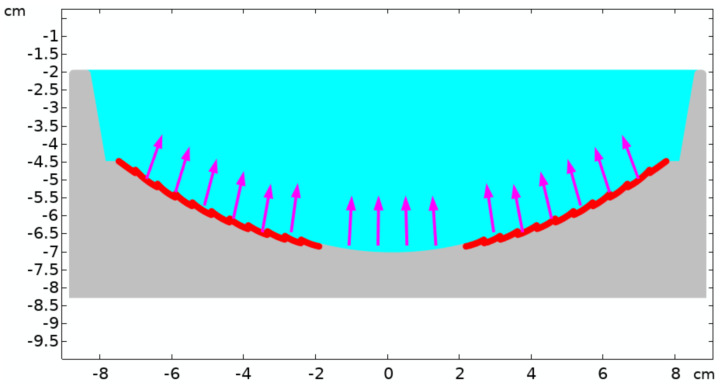
A thousand particles, each with a diameter of d=350 μm and density of $\rho =1.2\;g/{\mathrm{cm}}^{3}$ are submerged in water (colored in cyan) and are initially uniformly distributed across the curved bottom, as shown in the red circles. It is assumed that after the weights impact the crater, the crater descends and then rebounds, striking the particles and initiating their motion. Because of the concave shape of the bottom, the momentum transferred to the particles has both vertical and horizontal components, depending on their initial positions, as indicated by the arrows.

The behavior of the microspheres (called particles in the code) in the experiment was modeled using COMSOL^®^ Multiphysics software (version 5.5, COMSOL AB: Stockholm, Sweden) [32]. The simulation geometry shown in Figure 4 duplicates the experimental model exactly, except that the simulations are conducted in 2D.

Specifically, the horizontal velocity component of each particle is $V_{0}\sin\left(2\alpha \right)$ and the vertical component is $V_{0}\left(1+\cos\left(2\alpha \right)\right)$, where $V_{0}$ is the crater rebound velocity and sinα=x/R, where *x* is the particle horizontal position at the impact and *R* is the radius of the concave cavity. The exact value of the rebound velocity $V_{0}$ is not known but is likely a fraction of the crater impact velocity $V_{c}$. To investigate the effect of the rebound velocity, it was varied from 0.02 m/s to 5.0 m/s.

The equation of motion of each particle is governed by gravity, buoyancy, and viscous friction, and for a low Reynolds number (Re=ρdv/μ) (Stokes regime) it is:

(2) $$m\frac{d\vec{v}}{dt}=m\vec{g}\;\frac{\rho -\rho_{w}}{\rho }-m\frac{\vec{v}}{\tau_{\mu }},$$

where *m*, ρ, *d* and *v*, are the particle mass, density, diameter and velocity, and

(3) $$\tau_{\mu }=\frac{\rho d^{2}}{18\mu },$$

where μ is the water viscosity, is the characteristic viscous time.

A numerical estimate for the viscous time using $\rho =1.2\;g/{cm}^{3}$, d=350 μm, and $\mu =1\times 10^{-3}\mathrm{Pa}s$ gives $\tau_{\mu }=8.2\;ms$. The short viscous time means that, after an initial impulse, a particle comes to a standstill in just a few milliseconds. An approximate calculation of the distance traveled by a particle with an initial velocity of 0.5 m/s is l=4 mm, suggesting that traveling from the cavity edge at r=8 cm to the center requires at least 20 jolts or even more if only a fraction of the velocity is directed horizontally and gravity-induced deviations from horizontal motion are considered.

**Figure 5:**
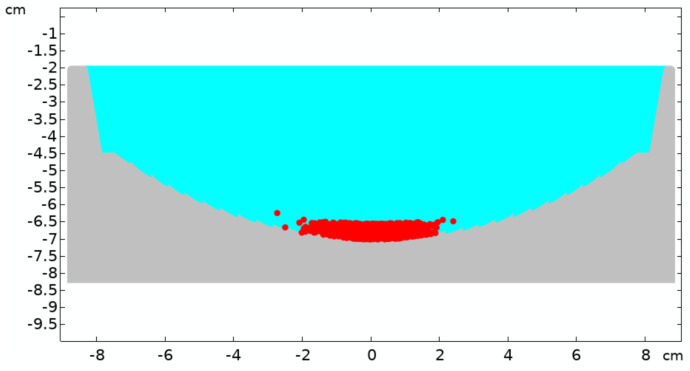
Particle distribution over the bottom of the cavity after 60 impacts at a rebound velocity of 0.5 m/s.

**Figure 6:**
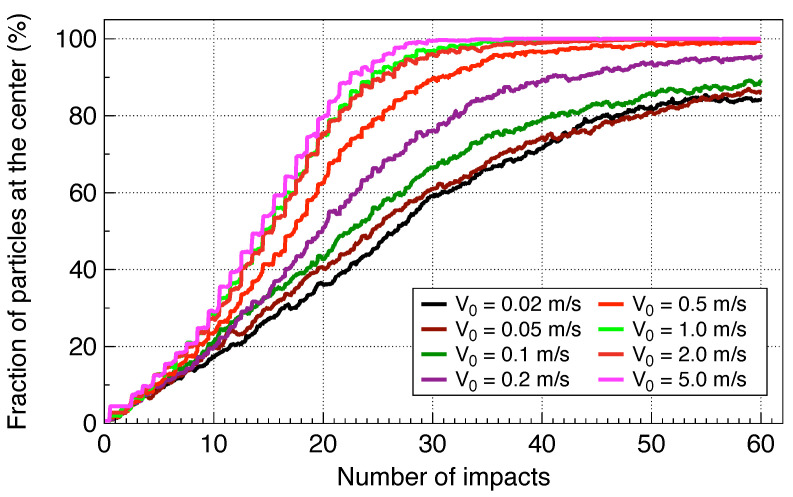
Evolution of particle concentration at the center at various rebound velocities, $V_{0}$. Shown is the time dependence of the fraction of the particle at the central area of the cavity r<2 cm. The small wiggles correspond to brief movements toward the center following each impact.

To extend the simulations over a broad Reynolds number range, the “standard drag correlation” (SDC) correction to the Stokes regime was applied. Figure 5 shows the particle distribution over the bottom of the cavity after consecutive 60 impacts at a rebound velocity of 0.5 m/s. A clear concentration enhancement is visible in the central area of the cavity. To explore the effect of the rebound velocity, it was varied from 0.02 m/s to 5.0 m/s. The result shown in Figure 6 confirms the quantitative estimates for the number of impacts needed. Indeed, according to the simulations, collecting 70% of all particles at a rebound velocity of $V_{0}=0.5\;m/s$ takes about 20 impacts, while reaching 90% of all the particles requires approximately 30 impacts, which aligns well with the estimates. Overall, the numerical results are in reasonable agreement with the experimental data.

One of the challenges this problem presents is scaling the presented results to the “real-life” liposome parameters. The primary scaling factor seems to be the liposome size. As shown by Equation (3), the characteristic time is inversely proportional to the square of the diameter. To illustrate this, reducing the liposome diameter by three orders of magnitude, from the 350 μm size used in simulations to a more realistic 350 nm, would increase simulation and experimental times by six orders of magnitude. Another challenge is accounting for fluid motion that our simulation does not include but could be significant, particularly at smaller particle sizes.

## 4. Summary and Conclusions

Various in vitro studies have demonstrated that upon physical contact, liposomes tend to fuse, grow, exchange contents, and divide. We hypothesize that gravity-driven and earthquake-assisted movement of liposomes within meteoric craters on Early Earth could lead to their accumulation at the crater floor. The paper presents both experimental and numerical investigations that demonstrate this phenomenon.

The “crater” for this experiment has a spherical cap shape with a sphere radius of 200 mm and a cap radius and height of 80 mm and 35 mm, respectively, and it was printed using water-tight 3D printing technology. The base of the crater features ridges spaced every 5 mm, each measuring 2.5 mm in height. The crater is filled with water into which fluorescent red plastic “liposomes”, with a diameter of d=350 μm and density of $\rho =1.2g\;/{\mathrm{cm}}^{3}$, are submerged. Clearly, neither the size and shape of the “crater” nor the size, material, and mass of the “liposomes” correspond to their real-life counterparts. Rather, the purpose of this experiment is a demonstration of a general principle of liquid-submerged micro-particulate dynamics, driven by gravitation and assisted by periodic disturbances.

The proposed vesicle congregation in meteoric craters does not constitute a definitive requirement for Darwinian evolution. Nevertheless, we see this hypothesis as a contributing factor in the population’s formation. This hypothesis satisfies at least three key prerequisites. First, the crater environment might contain primordial amphiphiles, phospholipid precursors, and ribose, which could have been either delivered by meteorites or synthesized there by the meteor impact. Second, filled with mineral-rich water, it provides vital protection against solar UV radiation. Third, soil movement aids in concentrating biomaterials at the crater’s center and encourages population formation. The experimental and simulation results presented here support the third prerequisite and demonstrate a proof of concept for this hypothesis.

## Data Availability

The original contributions presented in this study are included in the article. Further inquiries can be directed to the corresponding author.

## References

[1] Subbotin V., Fiksel G. (2023). Exploring the lipid world hypothesis: A novel scenario of self-sustained Darwinian evolution of the liposomes. Astrobiology.

[2] Subbotin V., Fiksel G. (2023). Aquatic ferrous solutions of prebiotic mineral salts as strong UV protectants and possible loci of life origin. Astrobiology.

[3] Turner B., Wu K., Fiksel G., Subbotin V. (2025). Protection of liposomes by ferric salts against the UV damage and its implications for the origin of life. Front. Astron. Space Sci..

[4] Adamski P., Eleveld M., Sood A., Kun Á., Szilágyi A., Czárán T., Szathmáry E., Otto S. (2020). From self-replication to replicator systems en route to de novo life. Nat. Rev. Chem..

[5] Mariano M.S., Fontanari J.F. (2024). Evolutionary game-theoretic approach to the population dynamics of early replicators. Life.

[6] Okasha S. (2022). The major transitions in evolution—A philosophy-of-science perspective. Front. Ecol. Evol..

[7] Takeuchi N., Hogeweg P. (2009). Multilevel selection in models of prebiotic evolution II: A direct comparison of compartmentalization and spatial self-organization. PLoS Comput. Biol..

[8] Das S., Pal R., Rajamani S. (2025). Dynamical interactions among protocell populations: Implications for membrane-mediated chemical evolution. Philos. Trans. R. Soc. B Biol. Sci..

[9] Mishra A., Taylor H., Patil A.J., Mann S. (2025). Dynamic Co-Clustering and Self-Sorting in Interactive Protocell Populations. Angew. Chem..

[10] Connor J., Yatvin M.B., Huang L. (1984). pH-sensitive liposomes: Acid-induced liposome fusion. Proc. Natl. Acad. Sci. USA.

[11] Deshpande S., Wunnava S., Hueting D., Dekker C. (2019). Membrane tension–mediated growth of liposomes. Small.

[12] Nir S., Bentz J., Wilschut J., Duzgunes N. (1983). Aggregation and fusion of phospholipid vesicles. Prog. Surf. Sci..

[13] Noguchi H., Takasu M. (2001). Fusion pathways of vesicles: A Brownian dynamics simulation. J. Chem. Phys..

[14] Chan Y.H.M., van Lengerich B., Boxer S.G. (2008). Lipid-anchored DNA mediates vesicle fusion as observed by lipid and content mixing. Biointerphases.

[15] Hanczyc M.M., Fujikawa S.M., Szostak J.W. (2003). Experimental models of primitive cellular compartments: Encapsulation, growth, and division. Science.

[16] Hardy M.D., Yang J., Selimkhanov J., Cole C.M., Tsimring L.S., Devaraj N.K. (2015). Self-reproducing catalyst drives repeated phospholipid synthesis and membrane growth. Proc. Natl. Acad. Sci. USA.

[17] Yang P., Lipowsky R., Dimova R. (2009). Nanoparticle formation in giant vesicles: Synthesis in biomimetic compartments. Small.

[18] Deshpande S., Spoelstra W.K., van Doorn M., Kerssemakers J., Dekker C. (2018). Mechanical division of cell-sized liposomes. ACS Nano.

[19] Döbereiner H.G., Käs J., Noppl D., Sprenger I., Sackmann E. (1993). Budding and fission of vesicles. Biophys. J..

[20] Penič S., Mesarec L., Fošnarič M., Mrówczyńska L., Hägerstrand H., Kralj-Iglič V., Iglič A. (2020). Budding and fission of membrane vesicles: A mini review. Front. Phys..

[21] Deamer D.W., Pashley R. (1989). Amphiphilic components of the Murchison carbonaceous chondrite: Surface properties and membrane formation. Orig. Life Evol. Biosph..

[22] Dworkin J.P., Deamer D.W., Sandford S.A., Allamandola L.J. (2001). Self-assembling amphiphilic molecules: Synthesis in simulated interstellar/precometary ices. Proc. Natl. Acad. Sci. USA.

[23] Martins Z., Pasek M.A. (2024). Delivery of organic matter to the early Earth. Elements.

[24] Zhao J., Mimura K. (2025). Supply of phospholipid precursors and evolution sites on the early Earth by impact. Geochim. et Cosmochim. Acta.

[25] Abe S., Yoda I., Kobayashi K., Kebukawa Y. (2024). Gamma-ray-induced synthesis of sugars in meteorite parent bodies. ACS Earth Space Chem..

[26] Furukawa Y., Chikaraishi Y., Ohkouchi N., Ogawa N.O., Glavin D.P., Dworkin J.P., Abe C., Nakamura T. (2019). Extraterrestrial ribose and other sugars in primitive meteorites. Proc. Natl. Acad. Sci. USA.

[27] Ono C., Sunami S., Ishii Y., Kim H.-J., Kakegawa T., Benner S.A., Furukawa Y. (2024). Abiotic ribose synthesis under aqueous environments with various chemical conditions. Astrobiology.

[28] Paschek K., Kohler K., Pearce B.K.D., Lange K., Henning T.K., Trapp O., Pudritz R.E., Semenov D.A. (2022). Possible ribose synthesis in carbonaceous planetesimals. Life.

[29] Cockell C.S. (2006). The origin and emergence of life under impact bombardment. Philos. Trans. R. Soc. B.

[30] Osinski G., Cockell C.S., Pontefract A., Sapers H.M. (2020). The role of meteorite impacts in the origin of life. Astrobiology.

[31] Mohraz B., Sadek F., Naeim F. (2001). Earthquake Ground Motion and Response Spectra. The Seismic Design Handbook.

[32] COMSOL AB (2019). COMSOL, version 5.5.

